# Validation of the Application of Solid Contact Ion-Selective Electrode for Off-Body Sweat Ion Monitoring

**DOI:** 10.3390/bios12040229

**Published:** 2022-04-09

**Authors:** Huixin Liu, Zhen Gu, Yuan Liu, Xinxin Xiao, Guangli Xiu

**Affiliations:** 1 Shanghai Environmental Protection Key Laboratory for Environmental Standard and Risk Management of Chemical Pollutants, School of Resources & Environmental Engineering, East China University of Science & Technology, Shanghai 200237, China; liuhuixin@mail.ecust.edu.cn; 2 State Environmental Protection Key Laboratory of Environmental Risk Assessment and Control on Chemical Processes, School of Resources & Environmental Engineering, East China University of Science & Technology, Shanghai 200237, China; 3 Shanghai Institute of Pollution Control and Ecological Security, Shanghai 200092, China; 4 Department of Automation, School of Information Science and Engineering, East China University of Science & Technology, Shanghai 200237, China; guzhen@ecust.edu.cn; 5 COFCO Corporation, Chao Yang Men South St. No. 8, Beijng 100020, China; liuyuan1@cofco.com; 6 Department of Chemistry, Technical University of Denmark, 2800 Kongens Lyngby, Denmark

**Keywords:** ion-selective electrode, ICP-OES, sweat ion, off-body

## Abstract

The solid contact ion-selective electrode (ISE) is a promising skin-interfaced monitoring system for sweat ions. Despite a growing number of on-body usages of ISE with fancy new materials and device fabrications, there are very few reports attempting to validate ISE results with a gold standard technique. For this purpose, this work uses inductively coupled plasma-optical emission spectrometry (ICP-OES) as a reference technique to conduct a direct evaluation of the sweat sodium and potassium ion levels obtained by ISE in an off-body approach. Eight healthy male subjects were recruited to collect exercise-induced sweat. It was found that sweat sodium and potassium ions present a rather wide concentration range. The sweat sodium concentration did not vary greatly in an exercise period of half an hour, while the sweat potassium concentration typically decreased with exercise. Mineral drink intake had no clear impact on the sweat sodium level, but increased the sweat potassium level. A paired *t*-test and mean absolute relative difference (MARD) analysis, a method typically used for evaluating the performance of glucometers, was employed to compare the results of ISE and ICP-OES. The statistical analysis validated the feasibility of ISE for measuring sweat ions, although better accuracy is required. Our data suggests that overweight subjects are likely to possess a higher sweat sodium level.

## 1. Introduction

Nowadays, the field of diagnostics is shifting to decentralization and personalized healthcare. Wearable biosensors [1,2,3,4], working in a minimally invasive or non-invasive manner, are of great interest due to their painless operation, enabling real-time and continuous monitoring of key biomarkers. Sweat, secreted by sweat glands located in the epidermis [5] on occasions such as physical exercise, heating, iontophoresis, and stress, is a readily available and accessible biofluid containing chemicals including metabolites, ions, and proteins, etc. Sweat, 99% of which is water, is a slightly acidic fluid with a pH ranging from 4.0 to 6.8 [6]. The levels of sweat chemicals vary, and reflect physical conditions, environments, and even pathophysiological status. Sweat analysis regarding the levels of chloride and sodium ions has already been established as a useful diagnostic tool for cystic fibrosis [7,8,9], a genetic disease characterized by the buildup of sticky mucus in the lungs and digestive system, and other diseases involving electrolyte imbalance disorder. Sweat has thus been recognized as a versatile platform for analytical purposes [10].

However, the correlation between sweat ion levels and health state is not yet well established [11], mainly due to the lack of understanding of the correlation between the ion levels in the sweat and blood plasma. The increased levels of sweat sodium and chloride are believed to be accompanied by an elevated sweat generation rate [5]. The sweat sodium level is independent from that in the plasma, while the sweat potassium level is likely to be correlated with that in the plasma [5]. In a recent paper analyzing the results of a relatively small number of subjects (10 healthy subjects and 6 patients) by Vario et al., it was found that there was no correlation between the ions in exercise-produced sweat and plasma, while the only correlation found was between the potassium in plasma and pilocarpine iontophoresis-induced sweat [12]. Although the physiological roles of sweat ions remain unclear, the quantitation of ion levels and pH still holds interest as they could affect the performance of a multiplex sensor platform with biosensors utilizing enzymes or aptamers [3,13].

Potentiometric ion-selective electrodes (ISEs) based electrochemical sensors have largely been used for the measurement of ion levels [12,14]. Further, all-solid-state ISEs which can eliminate the previous version comprising an inner-filling solution are well-established [15,16,17]. All-solid-state ISEs can be easily scaled down to analyze a very small volume of samples in microliters. It is not surprising that there is a rapid growth of publications reporting on-body all-solid-state ISEs for real-time sweat ion analysis [3,18,19,20,21,22]. The detection mechanisms of wearable sweat sensors are capable of real-time analysis, given that the rationalized microfluidic channel is designed for constant sweat sampling. However, these measured results are typically not validated for accuracy and reliability by gold standard analytical techniques, such as ion-chromatography (IC) or inductively-coupled plasma mass spectrometry (ICP-MS) [14,23,24,25]. One possible reason for this is that these techniques, relying on large instrumentation, are not suitable for on-body measurement. Furthermore, sampling to obtain a large enough volume of sweat is not an easy task.

Herein, we propose that the measurement of sweat ions by inductively-coupled plasma-optical emission spectrometry (ICP-OES) to validate the results obtained from homemade ISEs is paramount and necessary. We recruited eight healthy male subjects for the collection of sweat generated following cycling exercise for the off-body measurement of sodium and potassium ions. The time-profiles of electrolytes with and without mineral drink intake were also obtained. Statistical methods were then employed in order to better understand the relationship between the results obtained by bespoke all-solid-state ISEs and the gold standard ICP-OES.

## 2. Materials and Methods

### 2.1. Reagents and Materials

Sodium ionophore X, valinomycin (potassium ionophore), tetrahydrofuran, bis(2-ethylhexyl) sebacate (DOS), sodium tetrakis[3,5-bis(trifluoromethyl)phenyl]borate (Na-TFPB), polyvinyl chloride (PVC, K-value: 68–65), sodium chloride (NaCl, 99.99%), potassium chloride (KCl, 99.99%), and poly(3,4-ethylenedioxythiophene) polystyrene sulfonate (PEDOT:PSS) conducting polymer solution were obtained from Sigma-Aldrich Inc. (St. Louis, MO, USA). An Ag/AgCl reference electrode wire was obtained from Shanghai Chuxi Industrial Co., Ltd. (Shanghai, China). Epoxy AB glue was purchased from Smooth-On Inc. (Macungie, PA, USA) to seal all the electronic items. All reagents were analytical grade and used as received. All solutions were prepared using ultrapure water (18.2 MΩ cm at 25 °C) from a Milli-Q system (Billerica, MA, USA).

### 2.2. Human Sweat Sampling and Storage

Human sweat samples were collected from eight healthy male volunteers during spinning spiking. Prior to the investigation, all volunteers signed a permission form. The basic health information of all volunteers can be found in the supporting information (Table S1 in Supplementary Materials). Body mass index (BMI) was calculated using the following equation:

(1) $$BMI=\frac{Weight}{Height^{2}}$$

where weight is in kg and height is in meters.

**Scheme 1:**
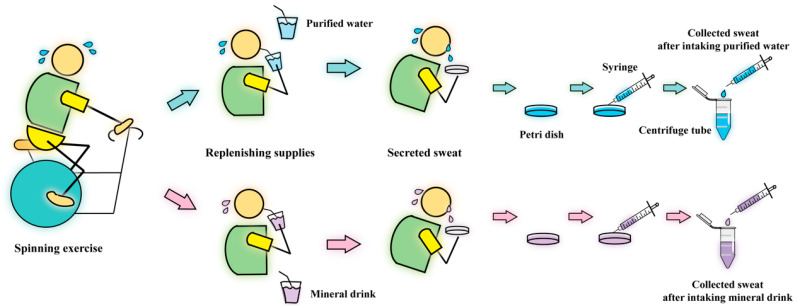
Schematic illustration of sweat collection procedures with intaking purified water or mineral drink.

Volunteers washed their faces with distilled water to avoid sample contamination in advance. Sampling was conducted in the evening at ca. 8 p.m., two hours after dinner. The room temperature was kept at 30 °C by air conditioning. During spinning, a Petri dish was placed under the chin to gather dripping sweat, which was then transferred into clean centrifuge tubes (Scheme 1). A 0.5 mL sweat sample was collected every 5 min, six times over half an hour. There were cases where the number of samples was less than six as the volunteer could not secret 0.5 mL sweat every 5 min. The sweat samples were all well sealed with Parafilm^®^ sealing film and stored at 4 °C before measurement.

Mineral drink intake during exercise has been considered as a key factor. For the control group, all volunteers drank 200–300 mL water 15 min before spiking, and, during the exercising process, ingested approximately 100 mL water every 10 min to keep a high sweat level. For the comparison group, all volunteers drank a mineral drink (bought in a local shop) with the same volume and time interval as the control group. According to the provided ingredient list, the concentrations of sodium and potassium ions in the mineral drink were up to 0.49 mg mL^−1^ and 0.21 mg mL^−1^, respectively. The levels of other electrolytes are listed in Table S2 (Supplementary Materials).

### 2.3. Sweat Samples Detection by the Designed Electrochemical Sensor

The details, including the selectivity coefficients, of the designed all-solid-state ISE-based sodium and potassium sensors have been documented in our previous work [24]. Briefly, the designed sensor is composed of a chemical sensor and a hardware section. The ion-selective membrane (ISM) modified sensor is fabricated on a printed circuit board (PCB), with soldered electronic items on the back. Through electroless nickel immersion gold (ENIG) technology, a gold layer is formed on the PCB and treated as the electrode layer to be modified to form PEDOT/PSS and ISM layers successively. A commercial Ag/AgCl electrode wire was used as the reference electrode. The collected potential values of the sodium and potassium ions from the designed sensor were converted to analyte concentrations according to the obtained calibration curve.

### 2.4. Sweat Sample Detection by ICP-OES

To show the accuracy and reliability of the results obtained by the proposed ISEs, the measurement of electrolyte concentrations from the same sweat samples was also tested by ICP-OES (Varian 710) with a relative standard deviation (RSD) within ±3%. Before measurement, sweat samples were diluted 500–1000 times to within the linear range of ICP-OES for sweat sodium and potassium.

### 2.5. Data Analysis

Data are reported as mean  ±  standard error (SD). For comparison of the two groups of data measured by the ISE and ICP-OES, paired two-tailed student’s *t*-tests and mean absolute relative difference (MARD) analyses were performed.

## 3. Results and Discussion

The developed ISEs exhibit linear detection ranges of 1.67 × 10^−3^–10^3^ and 7.96 × 10^−3^–10^3^ mM for the sodium and potassium ions, respectively [24], covering their concentration ranges in human sweat. Thus, the prepared ISEs can be used directly for sweat sodium and potassium measurement, without pre-dilution of the sweat samples. In comparison, dilution of the sweat samples by a factor of 500–1000 is required for measurement by ICP-OES, which features a very high sensitivity but a narrow detection range. The sweat collection method employed here was gathering the dripping sweat generated on the face during spinning exercises via Petri dishes before transfer to centrifuge tubes through syringes. Although this method is classical and likely to be influenced by evaporation or other potential issues, it provides a direct and simple way to obtain relatively pure sweat samples. The sodium and potassium ion concentrations of the sweat samples (*n* = 90) from the eight volunteers were measured and compared with both ISE and ICP-OES techniques.

**Figure 1:**
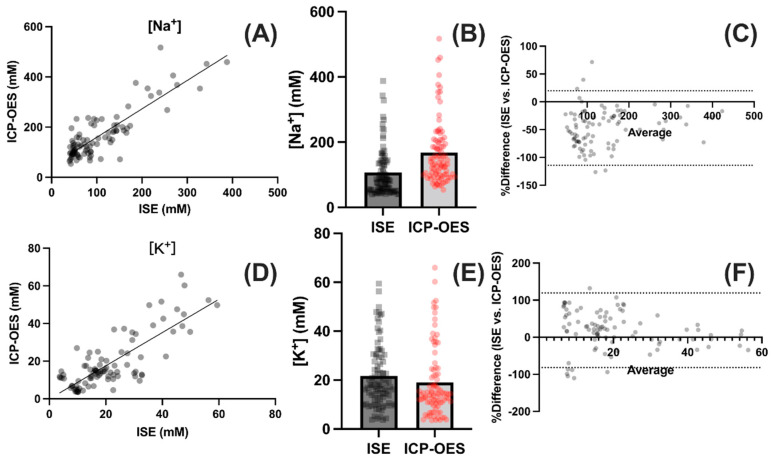
Comparison of sweat sodium (**A**–**C**) or potassium (**D**–**F**) ion level measured by ISE and ICP-OES. (**A**,**D**) Correlation of measured values of the two techniques. (**B**,**E**) Point plot of all the 90 independent measurements. (**C**,**F**) Bland Altman comparison plot of percentage of difference vs. average.

Figure 1A,D provides the correlation between the values obtained for sodium and potassium ions, respectively, generated by the ISE analysis and the ICP-OES method. Based on ICP-OES, the sodium and potassium ions were present in the sweat in rather wide ranges of 53.5–517.2 and 3.6–66.0 mM, with a mean ± SD of 167.6 ± 97.8 (Figure 1B) and 19.0 ± 14.2 mM (Figure 1E), respectively. The recorded upper limits are typically larger than those in the literature of 160 mM for Na^+^ and 30 mM for K^+^ for a healthy male [19], or 33.5 mM for Na^+^ and 23 mM for K^+^ for a healthy male [26]. The obtained mean values are notably higher than other previous reports of 56.7 ± 28.9 mM for Na^+^ and 4.5 ± 1.6 mM for K^+^ as collected from the foreheads of ten healthy males [27], and 30.9 ± 1.9 mM for Na^+^ and 12.2 ± 5.0 mM for K^+^ from ten healthy males [26]. These results reveal that the concentrations of sweat ions can vary extensively, requiring the designed ISE to have a rather wide linear detection range.

During validation of the recording of ISEs ([Na^+^]: 107.1 ± 74.1 mM; [K^+^]: 21.7 ± 12.9 mM; *n* = 90) by ICP-OES ([Na^+^]: 167.6 ± 97.8 mM; [K^+^]: 19.0 ± 14.2 mM; *n* = 90) (Figure 1B,E), a rather good correlation between the two techniques was found as confirmed by a paired *t*-test, registering a correlation coefficient (*r*) of 0.8569 for sodium ions and 0.8013 for potassium ions, respectively (Figure 1A,D). To better visualize the difference between the two groups of data, Bland–Altman comparison plots were further prepared (Figure 1C,F), providing the extend of the offsets, i.e., percentage difference, of all the measurements made by the two analytical methods. Most data points fell in the range of ±100%.

To perform a better comparison of the two techniques, we employed MARD analysis on the measured values, which is a frequently used statistical tool to characterize the analytical performance of continuous glucose monitoring systems [28]. MARD can be obtained by the comparison of the proposed meter (ISE in this case) and the reference meter (ICP-OES in this case) [29]:

(2) $$\mathrm{MARD}=\frac{1}{N}\overset{N}{\underset{k=1}{\sum }}\frac{\left|y_{ISE\left(k\right)}-y_{\text{ICP-OES}\left(k\right)}\right|}{y_{\text{ICP-OES}\left(k\right)}}$$

where *y_ISE_* and *y_ICP-OES_* represent the corresponding measured values by ISE and ICP-OES, respectively. MARD values were determined to be 39% for Na^+^ and 59% for K^+^. Overall, it can be concluded that the designed ISE can be used for revealing the real-time levels of sodium and potassium ions, as validated by ICP-OES, although a much higher accuracy is required for the proposed ISE.

**Figure 2:**
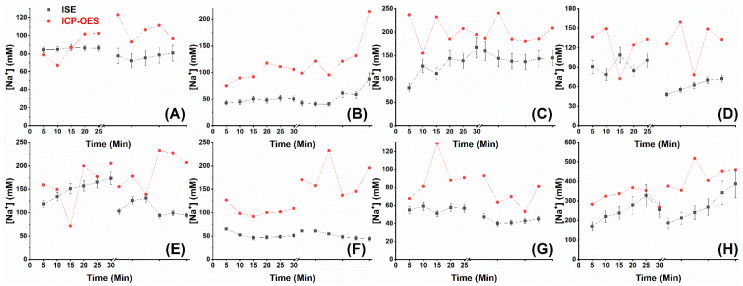
Time profile of the sweat sodium ions of eight volunteers (**A**–**H**). The data points on the left of the break on the X-axis are for the values collected for drinking purified water; while the data points on the right were collected for drinking a mineral drink. The time intervals between data points are the same in both sides of the break.

**Figure 3:**
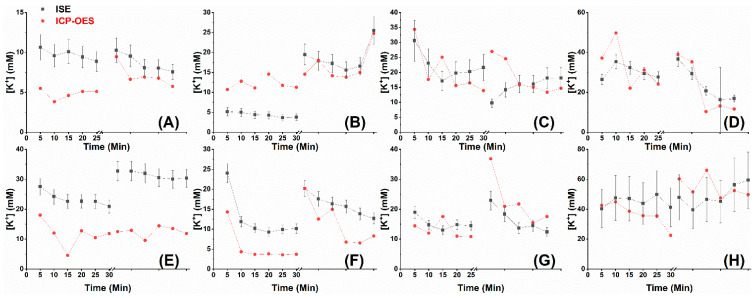
Time profile of the sweat potassium ions of eight volunteers (**A**–**H**). The data points on the left of the break on the X-axis are for the values collected for drinking purified water; while the data points on the right were collected for drinking a mineral drink. The time intervals between data points are the same in both sides of the break.

The individual time profiles of Na^+^ (Figure 2) and K^+^ (Figure 3) of the eight volunteers were then plotted. Despite the discrepancies, the tendencies of the time development of [Na^+^] and [K^+^] by ISE and ICP-OES are generally consistent with each other. The time profile of Na^+^ of each volunteer did not necessarily follow the same tendency of decreasing in a duration of 30 min (Figure 2), with volunteers A, B, F, and G maintaining a constant or slightly increased Na^+^ level with time, while [K^+^] typically showed a gradual decrease trend with time for all eight volunteers. Similar trends for [Na^+^] and [K^+^] have been observed in previously reported on-body tests [18,19]. [Na^+^] is believed to be positively correlated with sweat rate [27,30], and its increase indicates a rise of sweat secretion speed under intensive spinning.

We then assessed the effect of mineral drink intake, which contains electrolytes including sodium and potassium (Table S2 in Supplementary Materials) and is believed to supplement the body’s internal electrolytes [31]. It was found that mineral drink intake (Figure 2, data points on the left of the break on the X-axis) did not change [Na^+^] greatly, in comparison to the control of drinking purified water (Figure 2, data points on the right of the break on the X-axis). In contrast, [K^+^] was enhanced after drinking the mineral drink (Figure 3), which was obvious for volunteers A, F, and G. We believe this phenomenon is consistent with the above observation that [Na^+^] is not greatly consumed in an exercise period of 30 min, while [K^+^] is consumed in the same duration. Thus, supplement electrolytes have a clear impact towards [K^+^], rather than [Na^+^].

**Figure 4:**
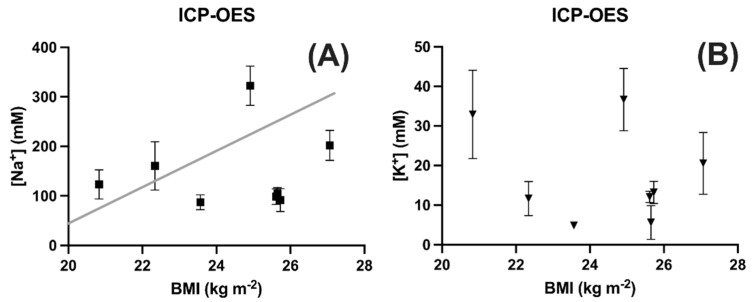
Correlation between body mass index (BMI) and sweat sodium (**A**) and potassium (**B**) ion level obtained via ICP-OES. The data are displayed as the mean ± SD obtained during the 30 min course for drinking purified water. The straight line in (**A**) is just a guide for the eye, not from a linear fitting.

The BMI, a metric used for calculating anthropometric height/weight characteristics in adults and an imperfect approximation for obesity [32], of each volunteer was obtained and considered as a parameter for sweat ions. The WHO recommends a BMI cut-off point of 25 kg m^−2^, above which is classified as overweight, and the threshold could be lower for Asian populations [33]. It was found that [Na^+^] was very weakly and positively correlated with BMI (*r*: 0.1353; *p* > 0.05), while [K^+^] was very weakly and negatively correlated with BMI (*r*: −0.2409; *p* > 0.05) (Figure 4), but these results were not statistically significant. It has been observed that obese subjects with high BMIs show significantly higher sweat rates than other groups [34], which can rationalize the positive correlation between [Na^+^] and BMI observed here (Figure 4A).

There is space for improvement in our approach: (i) the number of subjects could be expanded, particularly to include female subjects. In our initial efforts, it was found that females generally produce a lower volume of sweat than males, making it difficult to collect a suitable amount of sweat for analysis; (ii) improved sweat collection is expected when using more advanced tools or protocols [6,35,36], with negligible sweat loss, high purity, and simple operation; (iii) the sweat samples obtained here represent the accumulated sweat in a period of 5 min (the obtained ion concentration here is not measured in real-time). It is thus important to develop an on-body protocol to control the dead volume induced lag measurement, enabling small-volume and real-time monitoring. It is obvious that ISEs have such a potential for on-body measurement; (iv) the sweat samples were diluted 500–1000 times in order to be measurable by ICP-OES, which has a deviation within ±3%. Such a small deviation can be amplified to induce large errors. It is important to use a standard technique with a suitable detection range; and (v) the inevitable evaporation of sweat samples during storage should be taken into consideration, as it has been suggested to keep the sample at −80 °C [37]. Immediate measurement after sample collection is further suggested.

## 4. Conclusions

This work reports the validation of the usage of ISEs for sweat ions using the standard technique of ICP-OES. Based on the statistical analysis of a commonly-used paired *t*-test and MARD, an often-used method by the glucometer industry, it was confirmed that ISEs can be used for monitoring sweat sodium and potassium ions without sample pretreatment. Specifically, the registered correlation coefficient (*r*) of the two techniques is 0.8569 for sodium ions, and 0.8013 for potassium ions, respectively, with MARD values of 39% for sodium ions and 59% for potassium ions. The discrepancies between the two techniques are likely due to the fact that (i) the 500–1000-fold dilution of samples for ICP-OES may amplify errors; and (ii) there is an inevitable evaporation of the sweat samples during storage before measurement. Although a small number of subjects was studied here, the collected data implies that (i) sweat sodium and potassium ion levels vary in a considerably wide range; (ii) the time profile of sweat sodium does not change greatly in a period of half an hour, while sweat potassium shows a decreased level; (iii) sweat sodium level is not affected by mineral drink intake, while sweat potassium level is increased; and (iv) a weak and positive correlation between the sweat sodium level and BMI can be observed. Future work should focus on improving the sweat sampling method for both off-body and on-body measurements.

## Data Availability

Data available on request from the authors.

## Supplementary Materials

The following supporting information can be downloaded at: https://www.mdpi.com/article/10.3390/bios12040229/s1, Table S1: The specific health information of volunteers; Table S2: The ingredient list of chosen mineral drink as described in the label.
