# Supplementary material for: Validation of the Application of Solid Contact Ion-Selective Electrode for Off-Body Sweat Ion Monitoring
Source: Biosensors (Basel). 2022 Apr 9;12(4):229. doi: 10.3390/bios12040229 (PMC9026306; doi:10.3390/bios12040229)
Supplement: Supplementary file 1 [file biosensors-12-00229-s001.zip › biosensors-1656456-supplementary.pdf]

biosensors MDPI

Supplementary Materials

Validation of the Application of Solid Contact Ion-Selective Electrode for Off-Body Sweat Ion Monitoring

Huixin Liu [1,2,3], Zhen Gu [4], Yuan Liu [5], Xinxin Xiao [6,*] and Guangli Xiu [1,2,3,*]

[1] Shanghai Environmental Protection Key Laboratory for Environmental Standard and Risk Management of Chemical Pollutants, School of Resources & Environmental Engineering, East China University of Science & Technology, Shanghai 200237, China; liuhuixin@mail.ecust.edu.cn
[2] State Environmental Protection Key Laboratory of Environmental Risk Assessment and Control on Chemical Processes, School of Resources & Environmental Engineering, East China University of Science & Technology, Shanghai 200237, China
[3] Shanghai Institute of Pollution Control and Ecological Security, Shanghai 200092, China
[4] Department of Automation, School of Information Science and Engineering, East China University of Science & Technology, Shanghai 200237, China; guzhen@ecust.edu.cn
[5] COFCO Corporation, Chao Yang Men South St. No. 8, Beijng 100020, China; liuyuan1@cofco.com
[6] Department of Chemistry, Technical University of Denmark, 2800 Kongens Lyngby, Denmark
* Correspondence: xixiao@kemi.dtu.dk (X.X.); xiugl@ecust.edu.cn (G.X.)

Table S1. The specific health information of volunteers.

Volunteer	*Age*	*Gender*	*Height (m)*	*Weight (kg)*	*BMI (kg /m^{-2})*	*Health Status*
A	24	Male	1.75	73	23.57	Good
B	26	Male	1.80	83	25.62	Good
C	26	Male	1.73	81	27.06	Good
D	24	Male	1.82	69	20.83	Good
E	24	Male	1.82	74	22.34	Good
F	24	Male	1.71	75	25.65	Good
G	24	Male	1.73	77	25.73	Good
H	22	Male	1.70	72	24.91	Good

Table S2. The ingredient list of chosen mineral drink as described in the label.

**Ingredient**	**Content per 100 mL**
Sodium	49 (mg)
Potassium	21 (mg)
Energy	111 (kJ)
Protein	0 (g)
Fat	0 (g)
Carbohydrate	6.5 (g)
